# Psychometric Analyses of the Indian (Hindi) Version of the Child Perception Questionnaire (CPQ_11–14_)

**DOI:** 10.3390/children7100175

**Published:** 2020-10-09

**Authors:** Santosh Kumar Tadakamadla, Garima Mangal, Mir Faeq Ali Quadri, Maryam Nayeem, Jyothi Tadakamadla

**Affiliations:** 1 School of Dentistry and Oral Health, Griffith University, Gold Coast 4214, Australia; jyothi.tadakamadal@griffithuni.edu.au; 2 Buddha Dental College, Patna 800020, India; garima.mangal@gmail.com; 3 College of Dentistry, Jazan University, Jazan 45142, Saudi Arabia; dr.faeq.quadri@gmail.com (M.F.A.Q.); maryamnayeem12@gmail.com (M.N.)

**Keywords:** oral health-related quality of life, child perception questionnaire, validity, reliability, psychometric analysis

## Abstract

The current research aims to evaluate the reliability and validity of the Hindi Child Perception Questionnaire (CPQ_11–14_) in a child population of India. A randomly selected sample of children aged 11–14 years (*n* = 331) and their parents completed the Hindi translation of CPQ_11–14_ and the Parental-Caregiver Perceptions Questionnaire (P-CPQ), respectively, in this cross-sectional study. Children also provided a self-rating of oral health and were examined for dental caries. Exploratory Factor Analysis (EFA) was conducted to assess the dimensionality of the Hindi-CPQ_11–14_. Internal consistency and reliability on repeated administration were evaluated. Convergent and divergent validities were determined by estimating correlation coefficients between items and the hypothesised subscales. Concurrent validity was assessed using multiple linear regression analyses. The four factors extracted in EFA had a total variance of 38.5%, comprising 31 items. Cronbach’s alpha for the internal consistency of the overall scale was 0.90; reliability on repeated administration was 0.92. All the Hindi CPQ_11–14_ items had an item-hypothesised subscale correlation coefficient of ≥0.4, and these were greater than item-other hypothesised subscale correlations, demonstrating good convergent and divergent validities respectively. Hindi-CPQ_11–14_ was associated with self-ratings of the oral health and overall P-CPQ scores demonstrating good concurrent validity. Hindi-CPQ_11–14_ showed a factor structure different from the English CPQ_11–14_ and exhibited good validity and reliability.

## 1. Introduction

Oral diseases in children impact their day to day functions, such as eating, chewing, swallowing, speaking, and smiling [1,2,3]. Subsequently, they affect a child’s physical, emotional, social, and psychosocial wellbeing [4,5,6]. The assessment system investigating such impact of the oral condition on overall wellbeing has been termed Oral Health-Related Quality of Life (OHRQoL) [7,8]. Several child-specific OHRQoL assessment tools have been tested and utilised; the most common ones include Child-Oral Impact on Daily Performance (Child-OIDP) [9,10], Oral Health Impact Profile (Child-OHIP) [11,12], age-specific Child Perception Questionnaires (CPQ_8_–_10_ and CPQ_11–14_) [13], Parental-Caregiver Perceptions Questionnaire (P-CPQ) [14,15], and the Family Impact Scale [16]. 

Children residing in different parts of the world have a unique cultural identity and language, making them distinct from the children of other regions; thus, it is of the utmost importance to translate and adapt validated questionnaires for use in children from other cultures speaking different languages. This will not only assist in evaluating the region-specific burden of the psychosocial impact of poor oral health but also contribute to the global data on the OHRQoL of children. India is the second-largest populated country in the world. A popular language of the Indian sub-continent is Hindi, and a large population utilise Hindi as their only means of communication [17]. There are very few child-OHRQoL assessment tools that have been translated into Hindi and assessed for their psychometric properties [9,18,19,20]. One of the widely used age-specific OHRQoL assessment tools is CPQ_11–14_ [21], and its Hindi version has not yet been comprehensively tested and validated. The Department of School Education and Literacy under the umbrella of the Ministry of Human Resource and Development, Government of India, is actively working towards a proposal of “Three Language Formula”, that encourages the use of Hindi as a primary language to be taught in schools of India, with the other two languages being Urdu and one modern Indian language [22], thus considering the existing Hindi-speaking child population (approximately 40% of people in India speak Hindi [23]). Moreover, with the efforts taken to spread its use, a Hindi version of CPQ_11–14_ appears to be of great significance to enable the assessment of child-reported impact of oral health on overall quality of life. Besides this, the OHRQoL research in the child population of India is still emerging and requires a fair amount of work [13]. 

The original CPQ_11–14_ consists of 37 items that are organized into four subscales, namely oral symptoms, functional limitations, emotional wellbeing, and social wellbeing [14,24]. The previous attempt to validate the Hindi version of CPQ_11–14_ was not comprehensive; there were no attempts to assess the factor structure and dimensionality of the translated questionnaire [25]. Thus, the current study aimed to evaluate the reliability and validity of the cross-cultural adaptation of CPQ_11–14_ in the Hindi speaking child population of India.

## 2. Materials and Methods

### 2.1. Study Setting

This cross-sectional study was conducted among school children aged 11–14-years old in the Kankarbagh region of Patna City, India, during March to August 2016. Patna City is the capital of Bihar State, located in Northern India. It has a population of more than 1.6 million based on the 2011 census. It consists of 75 municipal wards belonging to five administrative circles, one of which is Kanakarbagh [26]. A cluster sampling technique was used, where a list of schools with Hindi as a medium of instruction was first obtained, and four schools (two were primary with grades up to 6 and two were high schools with grades starting from 7) were randomly selected through a simple random sampling procedure that have grades 5–8 corresponding to the 11–14 years age group. All the 11–14-year-old children (grades 5–8) in the selected schools were invited to participate by sending an information sheet and consent forms to the parents. Written informed consent was obtained from the parents and verbal consent from the children. Ethics approval (660/BIDSH) was obtained from the human research ethics committee of Buddha Dental College and permission was sought from school authorities. 

### 2.2. Data Collection

The data collection procedure involved administration of questionnaires to children and parents and clinical examination of children for dental caries. 

At first, the children completed the Hindi translation of CPQ_11–14_. The CPQ_11–14_ tool consists of 37 items with four subscales: oral symptoms (6 items), functional limitations (9 items), emotional wellbeing (9 items), and social wellbeing (13 items). The response scale for each item is on a five-point Likert scale with “Never” = 0; “Once/twice” = 1; “Sometimes” = 2; “Often” = 3; “Every day/almost every day” = 4. They also answered a question to self-rate their oral health status on a five-point Likert scale (response scale was 0 = excellent, 1 = very good, 2 = good, 3 = fair, and 4 = poor) [27]. In addition, the questionnaire comprised questions related to the socio-demographics and oral hygiene practices. Completion of questionnaire was followed by a clinical examination for dental caries in the permanent dentition by a single dental public health specialist. Dental caries were quantified using the Decayed, Missing, and Filled teeth (DMFT) index [28]. A tooth was considered carious if there was an unmistakable cavity, undermined enamel, and a softened floor or wall [29]. Clinical examinations were carried out in the classrooms under natural light using a plane mouth mirror and a Community Periodontal Index probe. Artificial light source was used when required. The examination for dental caries was repeated in 40 randomly selected children, and the intra-examiner reliability was assessed through kappa coefficient, which was 0.88. 

Consenting parents were then asked to complete a questionnaire sent to them through their children. Parent questionnaire comprised the Hindi translation of P-CPQ and questions related to socioeconomic status. P-CPQ is a 31-item proxy QoL instrument completed by parents. It has subscales and response scale similar to the CPQ_11–14_. A higher CPQ_11–14_ or P-CPQ score demonstrate poorer QoL. Family’s socioeconomic status was determined based on the Kuppuswamy scale using the level of education and occupation status of the head of the family along with the family income [30]. 

Repeated administration of the CPQ_11–14_ and P-CPQ was carried out after two weeks by providing the questionnaires to children and parents who agreed during the initial recruitment. All children and parents returned the completed questionnaires, and there were no missing data. 

### 2.3. Translation and Cross-Cultural Adaptation

Cross-cultural adaptation of CPQ_11–14_ [24], and P-CPQ [14] was undertaken using an established protocol [31]. English versions of the questionnaires were translated independently by two native Hindi speakers who were also fluent in English. A third translator compiled a questionnaire from the two translations, and any discrepancies between the translations were resolved by discussion. This was followed by a back-translation of the Hindi questionnaire to English by two translators independently who were fluent in both Hindi and English. A third translator compiled a single back-translated questionnaire. An expert committee composed of two researchers (fluent in both languages and public health experts) and three translators reviewed the two translated and back-translated versions to ensure the similarity of ideas and concepts between the original and translated questionnaires [32]. The translated questionnaires were then administered to 30 children and parents through interviews to maintain semantic equivalence. Minor modifications were made to three statements in the questionnaire for children. 

### 2.4. Statistical Analysis

Descriptive statistics were used to present the frequencies and means. An Exploratory Factor Analysis (EFA) using principal component analysis and varimax rotation (with Kaiser Normalisation) was conducted to assess the factor structure of the Hindi-CPQ_11–14_. Extraction was restricted to four factors to align with the dimensionality of the original instrument. Factorability of the items was assessed using Kaider–Meyer–Olkin (KMO) measure for sampling adequacy and Bartlett’s test of sphericity. A significant Barlett’s test of sphericity and a KMO score of >0.8 were considered as good indicators for factorability [33]. Internal consistency of the subscales and the overall scale was evaluated using Cronbach’s alpha while Intraclass Correlation Coefficient (ICC) was used to assess reliability on repeated administration. Cronbach’s alpha and ICC of 0.7 or higher were considered acceptable [34]. Pearson correlation coefficients were used to conduct the multi-trait scaling analysis, which involved an assessment of correlations between the items and hypothesised subscales. Convergent validity was supported if the item-hypothesised subscale correlation coefficient was 0.4 or higher, and discriminant validity was acceptable if the item-hypothesised subscale correlation was higher than the item’s correlation with other hypothesised subscales [35]. Correlations of the hypothesised subscale scores with the P-CPQ scale scores were also determined to assess convergent validity. Concurrent validity was evaluated using multiple linear regression analyses. Five multivariable models were constructed with the four hypothesised subscales and overall CPQ_11–14_ as dependent variables and self-rating of oral health, caries experience (DMFT), and total P-CPQ score as the independent variables after adjusting for age and sex. For the multivariable analyses, self-rating of oral health was dichotomised as 0 (good, very good, or excellent) and 1 (fair or poor). SPSS version 24.0 (IBM Corp, Armonk, New York, USA) was used to conduct the statistical analysis and a *p*-value of <0.05 was considered as significant. 

## 3. Results

From the invited participants (*n* = 380), 331 agreed and answered the survey, with a response rate of 87%. There were more boys (67.8%) than girls (32.2%). The mean age of the participants was 13.40 (standard deviation (SD): 1.12). Caries prevalence was very low (19.3%), with a mean DMFT of 0.39 (SD: 0.91). The majority (92.6%) of the children belonged to the middle (upper middle and lower middle of Kuppuswamy socioeconomic scale) socioeconomic class while there were no participants belonging to the high socioeconomic class. More than half of the children (59%) reported their oral health to be good, very good, or excellent.

In EFA, the total variance of 38.5% was explained by the four factors (eigenvalues of all four factors were >1). KMO measure of sampling adequacy was 0.89 and Bartlett’s test of sphericity was significant (*p* < 0.0001), confirming the factorability of the instrument. Table 1 demonstrates that six (“sores”, “bad breath”, “food between teeth”, “mouth breathing”, “felt nervous”, and “worried being different”) of the 37 items did not load on any of the factors and cross-loadings were minimal. Twelve items loaded on the first factor, while nine, six, and four items loaded on the second, third, and fourth factors, respectively. Based on the items that loaded on the first factor, it was named “oral symptoms, eating difficulties and school activities.” Item “irritated/frustrated” loaded on to the first factor while “argued” loaded on to the “social wellbeing” factor. All the items loading onto the second factor were related to “social wellbeing” while the third and fourth factors had “emotional wellbeing” and “functional items”, respectively. 

Factors “Oral symptoms, eating difficulties and school activities”, “Social wellbeing”, and “Emotional wellbeing” were found to have Cronbach’s alpha and ICC of >0.7. The factor “functional limitations” only had four items, with a Cronbach’s alpha of 0.59, while the ICC was 0.896. The Cronbach’s alpha for the overall scale was 0.90, while the ICC was 0.92 (Table 2). 

Table 3 demonstrates that most of the item-hypothesised subscale correlations were more than 0.4. The item-subscale correlation for “Oral symptoms, eating difficulties and school activities” ranged from 0.40 to 0.65 while the correlations for “social wellbeing” ranged from 0.58 to 0.72. “Emotional wellbeing” and “Functional limitations” had correlation coefficients in the range of 0.55–0.74 and 0.63–0.72, respectively. Moreover, all the items had higher correlation coefficients with their hypothesised subscale than the other subscales. Convergent validity of the subscales of the Hindi translation of CPQ was further confirmed through its correlation with their corresponding subscales of the P-CPQ. Table 4 shows that the strength of correlation of “Oral symptoms, eating difficulties and school” was high, with both “oral symptoms” (*r* = 0.54) and “functional limitations” (*r* = 0.51) subscales of the P-CPQ indicating that this scale comprises items related to both the subscales. The subscales of the Hindi-CPQ had the highest correlation with their corresponding P-CPQ subscales (r = 0.47 for social wellbeing and *r* = 0.43 for emotional wellbeing). 

‘Oral symptoms, eating difficulties and school’, ‘emotional wellbeing’ subscales and the overall scale were significantly associated with self-rating of oral health, with those reporting good/very good/excellent oral condition presenting lower scores (better QoL). However, caries status did not have any effect on the subscale or overall scores. All the subscale scores and overall CPQ score were positively associated with total P-CPQ scores, demonstrating that child-reported OHRQoL score increased as parent-reported children’s OHRQoL score increased (Table 5).

## 4. Discussion

Despite having a different factor structure from the original CPQ_11–14_, the 31-item Hindi-CPQ_11–14_ was found to be valid and reliable. Previously, OHRQoL in children was assessed by proxy questionnaires administered to parents, but parent- and child-reported impact of oral conditions on QoL had been found to vary [36]. Although parents’ knowledge of their children’s oral disease-related experiences is limited, they complement self-reported OHRQoL and provide a parent’s perspective [37]. However, the child-reported OHRQoL could give a more accurate depiction of the impact of the oral condition on their overall wellbeing. The current cross-sectional study assessed the dimensionality, reliability, and validity of the Hindi translation of CPQ_11–14_. The internal consistency reliability scores for some of the subscales and overall scale were comparable to the original version and other language versions which underwent cross-cultural adaptations [13,14]. However, the functional limitation subscale demonstrated poor internal consistency, and this could be due to there being very few items in this subscale. When there are less than five items in a subscale, a Cronbach’s alpha of 0.5–0.7 is also considered acceptable [38]. Contrastingly, the Telugu version [13] of the CPQ_11–14_ demonstrated higher ICC values than the original as well as the current version of the CPQ_11–14_. Slight differences in internal consistency and inter-examiner reliability values observed between our study and other studies could be due to the difference in the length of subscales or time intervals utilised. 

On conducting EFA, we found the factor structure of Hindi CPQ_11–14_ to be different from the original version, with only 31 items loading onto one of four factors [39,40]. The items related to school activities and eating, such as “school work”, “homework”, “eating difficulty”, and “biting difficulty” loaded onto the first factor, which had items related to the original “oral symptoms” subscale; which we renamed as “oral symptoms, eating difficulty, and school activities.” The reason could be that these items are an immediate consequence of persisting oral symptoms in our sample. The remaining items loaded onto the other three factors, emotional wellbeing, social wellbeing, and functional limitations. These factors corresponded to the factors in the original version of the CPQ_11–14_ [39]. The item “argued” loaded on the emotional wellbeing factor in contrast to social wellbeing, which was observed by the developers of the original CPQ_11–14_ [39]. This seems logical as arguing with someone would impact the emotional wellbeing of the individual rather than social wellbeing. Surprisingly, we also found that the item “felt unsure about yourself” loaded onto the “functional limitations” subscale along with three other items, “difficulty opening mouth, “difficulty saying words”, and “drinking with the straw”. Although the above three items are relevant to “functional limitations”, it was not clear why “felt unsure about yourself” loaded onto the same factor. This might be because of the negative impact of functional limitations on self-confidence [41]. 

Multiple linear regression analysis revealed that the self-rating of oral health was significantly associated with “Oral symptoms, eating difficulty, and school activities”, “emotional wellbeing”, and overall score. Correlations with self-ratings were observed in previous cultural adaptations of the CPQ_11–14_ [13,42]. However, none of the CPQ subscales were associated with the DMFT scores. We presume that this may be due to the fact that caries alone may not cause dysfunction (emotional, physical, schoolwork, etc.) unless they are accompanied with dental pain, and this result was observed to be consistent with another recent study [13]. However, Brown et al. [43] and Bhayat et al. [42] found a significant relationship between clinically examined oral health and all the subscale scores. This difference could be due to the variation in the severity of caries between the studies. We did not assess the extent of caries involvement (enamel, dentinal, or pulpal involvement) in this study. We used P-CPQ to evaluate convergent validity as it closely relates to CPQ_11–14_, and similar findings with the concurrent methods were observed in another recent report [44]. 

It is important to reiterate that very few published reports on the psychometric analysis of CPQ_11–14_ have investigated the relationship between the caries status and the subscales of CPQ_11–14_ after adjusting for confounders such as age and sex [45]. Another strength of this study was its use of the probability sampling technique to recruit representative children and parents. Additionally, we also conducted EFA to evaluate the dimensionality of the instrument in our population, which was not done in many past studies that conducted a cross-cultural adaptation of the CPQ_11–14_. Caries status was the only clinical evaluation done in this study; it would be interesting to see if other clinical parameters (gingival/periodontal status or malocclusion) would impact the QoL. Longitudinal studies will help to determine the responsiveness of the Hindi CPQ_11–14_ to change in clinical status.

## 5. Conclusions

The Hindi CPQ_11–14_ demonstrated a factor structure different from the original instrument and comprised 31 items. All the subscales and the overall scale demonstrated acceptable internal consistency and reliability on repeated administration. Hindi CPQ_11–14_ was found to have good convergent validity, as shown through the correlation of its subscales with the corresponding subscales of the P-CPQ. The subscales of the Hindi-CPQ_11–14_ were also associated with self-ratings of oral health and overall P-CPQ scores, demonstrating good concurrent validity. 

## Figures and Tables

**Table 1:** Factor loadings of the Hindi version of CPQ_11–14._

CPQ_11–14_ Items	Factors			
	1	2	3	4
Pain in teeth, lips, jaws or mouth	0.62			
Bleeding gums	0.45			
Food stuck to the roof of the mouth	0.52			
Taken longer than others to eat a meal	0.52			
Trouble sleeping	0.61			
Difficult to bite or chew food like apples, corn on the cob or steak	0.59			
Difficult to open your mouth wide				0.54
Difficult to say any words				0.57
Difficult to eat foods you would like to eat	0.53			
Difficult to drink with a straw				0.53
Difficult to drink or eat hot or cold foods	0.50			
Irritable/frustrated	0.42			
Felt unsure of yourself				0.53
Shy/embarrassed			0.60	
Concerned with what other people think			0.61	
Worried that is less attractive than other people			0.66	
Upset			0.56	
Worried that is less healthy than other people			0.47	
Missed school because of pain, appointment or surgery	0.40			
Had a hard time paying attention in school	0.60			
Had difficulty doing your homework	0.55			
Not wanted to speak/read out loud in class		0.56		
Not wanted/been unable to participate in sports, clubs		0.69		
Not wanted to talk to other children		0.63		
Avoided smiling/laughing when around other children		0.46		
Had difficulty playing a musical instrument such as a recorder, flute, clarinet, trumpet		0.57		
Not wanted to spend time with other children		0.55		
Argued with other children or your family			0.45	
Teased/called names by other children		0.50		
Left out by other children		0.68		
Asked questions about your teeth, lips, jaws or mouth by other children		0.51		

**Table 2:** Reliability statistics for the subscales and total scale of the Hindi translation of CPQ_11–14._

CPQ_11–14_ Subscales	Number of Items	Cronbach’s Alpha	ICC
Oral symptoms, eating difficulties and school activities	12	0.82	0.95 (0.92–0.97)
Social wellbeing	9	0.81	0.90 (0.85–0.94)
Emotional wellbeing	6	0.76	0.89 (0.82–0.93)
Functional limitations	4	0.59	0.90 (0.84–0.94)
CPQ_11–14_ overall scale	31	0.90	0.91 (0.85–0.95)

ICC = Intra-class Correlation Coefficient.

**Table 3:** Multi-trait scaling of the subscales of the Hindi version of CPQ_11–14._

	Items	Oral Symptoms, Eating Difficulties and School Activities	Social Wellbeing	Emotional Wellbeing	Functional Limitations
**Oral symptoms, eating difficulties and school activities**	Pain in teeth, lips, jaws or mouth	0.65 **	0.30 **	0.39 **	0.20 **
	Bleeding gums	0.54 **	0.26 **	0.28 **	0.20 **
	Food stuck to roof of mouth	0.59 **	0.26 **	0.33 **	0.28 **
	Taken longer than others to eat a meal	0.60 **	0.31 **	0.39 **	0.27 **
	Trouble sleeping	0.59 **	0.24 **	0.30 **	0.32 **
	Difficult to bite or chew food like apples, corn on the cob or steak	0.64 **	0.32 **	0.40 **	0.27 **
	Difficult to eat foods you would like to eat	0.60 **	0.32 **	0.35 **	0.33 **
	Difficult to drink or eat hot or cold foods	0.57 **	0.21 **	0.34 **	0.21 **
	Irritable/frustrated	0.61 **	0.35 **	0.47 **	0.38 **
	Missed school because of pain, appointment or surgery	0.40 **	0.26 **	0.13^*^	0.25 **
	Had hard time paying attention in school	0.63 **	0.34 **	0.33 **	0.26 **
	Had difficulty doing your homework	0.61 **	0.34 **	0.31 **	0.32 **
**Social wellbeing**	Not wanted to speak/read out loud in class	0.27 **	0.58 **	0.28 **	0.20 **
	Not wanted/been unable to participate in sports, clubs	0.29 **	0.69 **	0.34 **	0.25 **
	Not wanted to talk to other children	0.34 **	0.65 **	0.29 **	0.22 **
	Avoided smiling/laughing when around other children	0.28 **	0.58 **	0.35 **	0.26 **
	Had difficulty playing a musical instrument such as a recorder, flute, clarinet, trumpet	0.32 **	0.62 **	0.23 **	0.35 **
	Not wanted to spend time with other children	0.27 **	0.62 **	0.34 **	0.35 **
	Teased/called names by other children	0.29 **	0.61 **	0.43 **	0.30 **
	Left out by other children	0.36 **	0.72 **	0.44 **	0.29 **
	Asked questions about your teeth, lips, jaws or mouth by other children	0.41 **	0.65 **	0.49 **	0.34 **
**Emotional wellbeing**	Shy/embarrassed	0.39 **	0.45 **	0.73 **	0.34 **
	Concerned with what other people think	0.43 **	0.46 **	0.74 **	0.30 **
	Worried of being less attractive than other people	0.32 **	0.31 **	0.69 **	0.21 **
	Upset	0.50 **	0.36 **	0.68 **	0.40 **
	Worried of being less healthy than other people	0.38 **	0.28 **	0.61 **	0.32 **
	Argued with other children or your family	0.33 **	0.40 **	0.55 **	0.29 **
**Functional limitations**	Difficult to open your mouth wide	0.37 **	0.35 **	0.38 **	0.72 **
	Difficult to say any words	0.24 **	0.30 **	0.28 **	0.67 **
	Difficult to drink with a straw	0.32 **	0.21 **	0.20 **	0.63 **
	Felt unsure of yourself	0.33 **	0.34 **	0.36 **	0.65 **

** Correlation is significant at the 0.01 level (2-tailed).

**Table 4:** Correlation between the subscales of the Hindi translation of CPQ_11–14_ and P-CPQ demonstrating convergent validity.

Subscales CPQ	Oral Symptoms P-CPQ	Functional Limitations P-CPQ	Emotional Wellbeing P-CPQ	Social Wellbeing P-CPQ
Oral symptoms, eating difficulties and school	0.54 **	0.51 **	0.41 **	0.40 **
Social wellbeing	0.36 **	0.32 **	0.38 **	0.47 **
Emotional wellbeing	0.39 **	0.39 **	0.43 **	0.39 **
Functional limitations	0.24 **	0.29 **	0.30 **	0.41 **

** Correlation is significant at the 0.01 level (2-tailed).

**Table 5:** Multiple linear regression analysis with the subscale and overall Hindi CPQ_11–14_ scale as the dependent variables (adjusted for age and sex).

Variables	Oral Symptoms, Eating Difficulties and School		Social Wellbeing		Emotional Wellbeing		Functional Limitations		Total CPQ_11–14_ Score	
	B (SE)	95% CI	B (SE)	95% CI	B (SE)	95% CI	B (SE)	95% CI	B (SE)	95% CI
**Self-rating of oral health**										
Good, very good or excellent	−2.19 (0.58) *	−3.33, −1.05	−0.55 (0.52)	−1.57, 0.47	−1.31 (0.43) **	−2.14, −0.47	−0.12 (0.24)	−0.60, 0.36	−4.17 (1.32) *	−6.76, −1.58
Fair or poor	Reference		Reference		Reference		Reference		Reference	
**DMFT**	0.48 (0.29)	−0.09, 1.05	−0.17 (0.26)	−0.68, 0.35	−0.004 (0.21)	−0.42, 0.42	−0.05 (0.12)	−0.29, 0.19	0.26 (0.12)	−1.04, 1.56
**Total P-CPQ Score**	0.2 (0.02) **	0.15, 0.24	0.14 (0.02) **	0.10, 0.18	0.12 (0.02) **	0.08, 0.15	0.06 (0.01) **	0.04, 0.08	0.52 (0.05) **	0.41, 0.61

* *p* < 0.05, ** *p* < 0.01.
